# Early post-operative recovery of consciousness following decompressive craniectomy as a predictor of long-term functional outcome

**DOI:** 10.1016/j.bas.2025.104241

**Published:** 2025-03-20

**Authors:** Jari C. Göttgens, Taco Goedemans, Dagmar Verbaan, Bert A. Coert, Bertjan J. Kerklaan, Jonathan M. Coutinho, Janneke Horn, William P. Vandertop, Pepijn van den Munckhof

**Affiliations:** a Department of Neurosurgery, Amsterdam University Medical Centers, Location Academic Medical Center, Meibergdreef 9, 1105 AZ, Amsterdam, the Netherlands; b Department of Neurology, Onze Lieve Vrouwe Gasthuis, Location West, Jan Tooropstraat 164, 1061 AE, Amsterdam, the Netherlands; c Department of Neurology, Amsterdam University Medical Centers, Location Academic Medical Center, Meibergdreef 9, 1105 AZ, Amsterdam, the Netherlands; d Department of Intensive Care, Amsterdam University Medical Centers, Location Academic Medical Center, Meibergdreef 9, 1105 AZ, Amsterdam, the Netherlands

**Keywords:** Decompressive craniectomy, Consciousness, Outcome, Glasgow coma scale, Glasgow outcome scale

## Abstract

**Introduction:**

Decompressive craniectomy (DC) can be lifesaving, but many survivors do not regain independence in daily life. Recovery of consciousness in the first post-operative days is regarded as a prognostic factor, however, literature on the relation between early recovery of consciousness and long-term independence is scarce.

**Research question:**

To analyse the relation between recovery of consciousness in the first 14 days post-DC and long-term functional outcome.

**Material and methods:**

Glasgow Coma Scale (GCS) motor (M) scores during the first 14 post-DC days of 188 consecutive adult patients undergoing DC for various pathologies were retrospectively extracted from hospital records, together with one-year Glasgow Outcome Scale (GOS) scores. Recovery of consciousness was defined as GCS M6. Outcome was categorised into death (GOS 1), unfavourable survival (GOS 2–3), and favourable survival (GOS 4–5).

**Results:**

Overall, 32 % survived favourably, 21 % unfavourably, and 47 % died. One hundred and eight patients (57 %) regained consciousness during the first two post-operative weeks. At one year, 53 % of M6 patients were functionally independent, versus only 4 % of patients who did not regain consciousness during that time-frame (p < 0.001). Chances of functionally independent survival in M6 patients were significantly higher in patients ≤50 years old than in patients >50 years old (71 % versus 27 %, p < 0.001).

**Discussion and conclusion:**

Long-term functional outcome of DC patients differed considerably when assorted for early recovery of consciousness, especially when categorised for age. These results may serve to better inform family members and patients during post-DC counselling.

## Highlights

• Decompressive craniectomy (DC) can be lifesaving, but many do not regain independence.
• Recovery of consciousness in the first 14 post-DC days may predict long-term functional outcome.
• The relation between early recovery of consciousness and long-term functional outcome was analysed in 188 DC patients.
• 53 % of patients who regained consciousness survived functionally independent, versus only 4 % of patients who did not.
• 71 % of conscious patients ≤50 years survived functionally independent, versus only 27 % of patients >50 years old.

## 1 Introduction

Decompressive craniectomy (DC) can be lifesaving in patients with traumatic brain injury (TBI) or space-occupying cerebral infarction, but many survivors do not regain independence in activities of daily living (ADL) (Dower et al., 2022; Sahuquillo and Dennis, 2019). Neurological recovery in the first days after DC could be informative for long-term functional outcome: patients with swift recovery of consciousness are often regarded to have a better chance of regaining functional independence than patients who remain unconscious for a prolonged period. However, due to the mere absence of data in the current literature, this relation remains unclear, whereas such data could be highly valuable during post-DC counselling of family members and patients. To address this issue, we analysed recovery of consciousness in the first 14 days post-DC in a cohort of 188 consecutive DC patients, together with its relation with long-term functional outcome.

## 2 Methods

### 2.1 Patient population

This retrospective cohort included all adult patients from a pre-existing cohort which underwent DC in our university medical centre between January 1, 2006 and January 1, 2014 for traumatic brain injury (TBI), ischemic stroke, intracerebral haemorrhage (ICH), aneurysmal subarachnoid haemorrhage (SAH), cerebral venous thrombosis (CVT), or brain infection/tumour (other) (Goedemans et al., 2017). Formal institutional ethical review board approval for this observational study was waived, patients’ consent was not required.

### 2.2 Clinical management, decompressive craniectomy and post-operative course

Details of pre-operative management and used technique for DC have previously been described in detail (Goedemans et al., 2017). In brief, all patients underwent computed tomography (CT) imaging of the brain. Relevant intracranial masses were surgically evacuated. In some patients, DC was performed in the same surgery, in others secondary DC was performed during a subsequent operation when further neurological deterioration occurred or when intracranial pressure (ICP) elevations >25 mm Hg for longer than 1 h were observed, despite non-surgical interventions. DC consisted of the excision of a large bone flap, including the frontal, temporal, and parietal bones, with a diameter of at least 120 mm. Then wide opening of the dura was performed. The cortical surface was covered with the un-approximated dural flaps and absorbable haemostatic cellulose. After surgery, all patients were admitted to the intensive care for protocolled supportive treatment.

### 2.3 Data collection

Data on baseline demographics, aetiology of brain injury, pre-DC Glasgow Coma Scale (GCS) scores, length of intensive care unit (ICU) and hospital stay, and causes of death were extracted from the pre-existing database (Goedemans et al., 2017). With the GCS motor (M) score 6 being the most accurate post-DC monitored depicter of recovery of consciousness, data on GCS M-scores during the first 14 post-DC days were extracted from the hospital records, together with sedation status and additional surgeries (if applicable). When multiple GCS M-scores per post-DC day were registered, we included the highest score in the current analysis. When a patient was transferred to a referring hospital or rehabilitation clinic within the first 14 post-DC days, or when a patient died within this time frame, no further M-scores were collected. Clinician's notes at one-year post-DC were used to assess outcome according to the Glasgow Outcome Scale (GOS) (Jennett and Bond, 1975). When no one-year post-DC clinical notes were available in our centre, referring neurologists and general practitioners were consulted. The STROBE guidelines were used in order to report this observational study accurately and completely (von Elm et al., 2008).

### 2.4 Data analysis

Death was defined as GOS score 1, unfavourable survival as GOS scores 2–3 (persistent vegetative state or severe disability, i.e. dependence in ADL), and favourable survival as GOS scores 4–5 (moderate or low disability, i.e. independence in ADL). As shown in our previous work, age was a significant predictor of outcome (Goedemans et al., 2017). The relation between post-DC GCS M-scores and long-term functional outcome was therefore analysed for the age groups younger and older than the median age of 51 years old (≤50 versus >50 years old). Continuous variables were tested for normal distribution using the Shapiro-Wilk test. A variable was considered normally distributed if the Shapiro-Wilk test was >0.9. Means (±standard deviation, SD) are given for normally distributed continuous variables whereas median (interquartile range, IQR 25 %–75 %) are given for not normally distributed continuous variables. Fisher exact test (for analysis of 2 × 2 tables) and chi-square test (for analysis of N x 2 contingency tables) were done as appropriate to identify differences between outcome groups. IBM SPSS Statistics 28.0 was used for calculations.

## 3 Results

Our previous study included 204 consecutive patients undergoing DC (Goedemans et al., 2017). In four patients, no post-operative clinical notes were found in the hospital archives. Furthermore, 12 patients were under the age of 18 years old. The current study therefore included 188 patients. Baseline characteristics are presented in Table 1. Overall, 60 patients (32 %) survived favourably (17 GOS-5, 43 GOS-4), 40 (21 %) unfavourably (38 GOS-3, 2 GOS-2), and 88 patients (47 %) died. In patients ≤50 years old, favourable survival was significantly higher than in patients >50 years old (51 % versus 14 %, p < 0.001), whereas mortality was significantly lower (31 % versus 62 %, p < 0.001), while the percentage of patients surviving unfavourably was similar (18 % versus 24 %, p = 0.21).

**Table 1:** Characteristics of 188 patients undergoing decompressive craniectomy for various pathologies categorised by one-year outcome.

	Total cohort	Death	Unfavourable survival	Favourable survival
n (male/female)	188 (97/91)	88 (51/37)	40 (19/21)	60 (27/33)
Age (yrs) median (IQR), range	51 (40–59), 18-78	57 (45–64), 18-78	52 (44–58), 23-71	43 (34–49), 19-68
Age 18–50 yrs, n (%)	93 (49)	29 (31)	17 (18)	47 (51)
Age 51–78 yrs, n (%)	95 (51)	59 (62)	23 (24)	13 (14)
Indication for DC				
Ischemic stroke, n (%)	69 (37)	26 (38)	22 (32)	21 (30)
TBI, n (%)	43 (23)	28 (65)	5 (12)	10 (23)
SAH, n (%)	28 (15)	12 (43)	8 (29)	8 (29)
ICH, n (%)	25 (13)	16 (64)	2 (8)	7 (28)
CVT, n (%)	14 (7)	3 (21)	1 (7)	10 (71)
Other, n (%)	9 (5)	3 (33)	2 (22)	4 (44)
Neurologic condition pre-DC				
M6, n (%)	32 (17)	6 (19)	11 (34)	15 (47)
M5, n (%)	39 (21)	13 (33)	8 (21)	18 (46)
M ≤ 4, n(%)	32 (17)	17 (53)	7 (22)	8 (25)
Intubated & sedated, n (%)	83 (44)	51 (61)	14 (17)	18 (22)
Unknown, n (%)	2 (1)	1 (50)	0	1 (50)
Additional surgery after DC, n (%)	56 (30)	28 (50)	16 (29)	12 (21)
Best neurologic condition first 14 days post-DC				
M6, n (%)	108 (57)	25 (23)	26 (24)	57 (53)
M5, n (%)	24 (13)	10 (42)	12 (50)	2 (8)
M ≤ 4, n (%)	56 (30)	53 (95)	2 (4)	1 (2)
ICU stay, days, median (IQR)	5 (2–12)	6 (3–13)	8 (3–18)	3 (1–7)
Hospital stay, days, median (IQR)	25 (9–45)	9 (4–27)	49 (28–79)	27 (19–40)
Cranioplasty within 1 year, n (%)	81 (43)	3 (4)	28 (35)	50 (61)

CVT: cerebral venous thrombosis; DC: decompressive craniectomy; ICH: intracerebral hematoma; ICU: intensive care unit; IQR: interquartile range; M: Glasgow Coma Scale motor score; n: number of patients; PO1: post-operative day 1; SAH: aneurysmal subarachnoid haemorrhage; SD: standard deviation; TBI: traumatic brain injury; yrs: years.

### 3.1 Outcome in patients regaining consciousness in the first 14 days post-DC

**Fig. 1:**
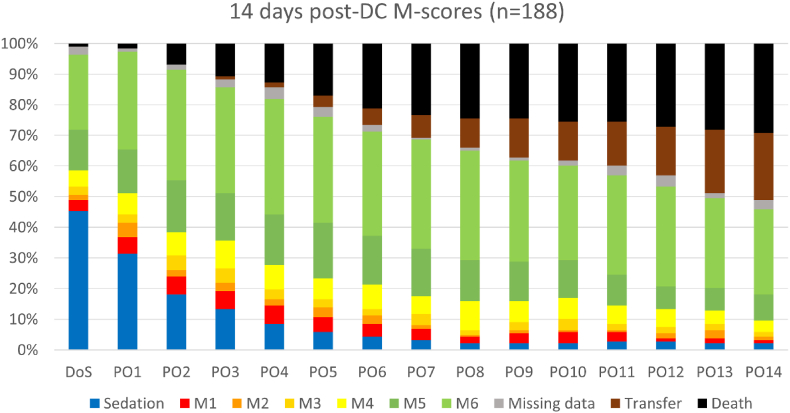
Post-operative course of Glasgow Coma Scale motor scores during the first 14 days after decompressive craniectomy (DC) of 188 consecutive adult patients undergoing DC for various pathologies. DoS, day of surgery; M, Glasgow Coma Scale motor score; n, number of patients; PO, post-operative day; Sedation, sedated patients without recorded motor score; Transfer, patients transferred to referring hospitals or rehabilitation clinics.

Fig. 1 displays the recorded GCS scores during the first 14 days post-DC. One hundred and eight patients (57 %) scored M6 during this time-frame. Their baseline characteristics are presented in Table 2. At post-DC day 1, 60 patients (32 %) were awake. At post-DC day 7, 68 patients (36 %) were awake, while another 10 awake patients (5 %) had been transferred to referring hospitals or rehabilitation clinics before that day. At post-DC day 14, 52 patients (28 %) were awake, while 34 awake patients (18 %) had been transferred before that day. After regaining consciousness, 34 patients remained awake and admitted up to post-DC day 14, 28 awake patients were transferred to referring hospitals or rehabilitation clinics, whereas 45 patients experienced subsequent neurological deterioration (by post-DC day 14, 20 out of 45 patients had restored consciousness again). In one awake patient at post-DC day 7, no further GCS scores could be retrieved from the clinical files.

**Table 2:** Characteristics of 108 patients undergoing decompressive craniectomy scoring GCS M6 during the first 14 post-operative days, categorised by one-year outcome.

	M6 cohort	Death	Unfavourable survival	Favourable survival
n (male/female)	108 (52/56)	25 (14/11)	26 (14/12)	57 (24/33)
Age (yrs) median (IQR), range	51 (40–59), 18-78	57 (47–63), 30-77	52 (44–58), 23-66	44 (34–49), 24-68
Age 18–50 yrs, n (%)	63 (58)	7 (11)	11 (17)	45 (71)
Age 51–78 yrs, n (%)	45 (42)	18 (40)	15 (33)	12 (27)
Indication for DC				
Ischemic stroke, n (%)	47 (44)	8 (17)	18 (38)	21 (45)
TBI, n (%)	11 (10)	3 (27)	1 (9)	7 (64)
SAH, n (%)	17 (16)	5 (29)	4 (24)	8 (47)
ICH, n (%)	15 (14)	7 (47)	1 (7)	7 (47)
CVT, n (%)	11 (10)		1 (9)	10 (91)
Other, n (%)	7 (6)	2 (29)	1 (14)	4 (57)
Neurologic condition pre-DC				
M6, n (%)	30 (28)	4 (13)	11 (37)	15 (50)
M5, n (%)	31 (29)	6 (19)	7 (23)	18 (58)
M ≤ 4, n(%)	19 (18)	7 (37)	4 (21)	8 (42)
Intubated & sedated, n (%)	27 (25)	8 (30)	4 (15)	15 (56)
Unknown, n (%)	1 (1)			1 (100)
Additional surgery after DC, n (%)	30 (28)	11 (37)	9 (30)	10 (33)
Neurological deterioration following M6 in first 14 days post-DC	45 (42)	19 (42)	6 (13)	20 (44)
ICU stay, days, median (IQR)	5 (1–11)	12 (4–18)	6 (1–14)	2 (1–7)
Hospital stay, days, median (IQR)	30 (21–47)	32 (13–53)	40 (22–78)	27 (18–39)
Cranioplasty within 1 year, n (%)	71 (66)	2 (3)	20 (28)	49 (69)

CVT: cerebral venous thrombosis; DC: decompressive craniectomy; ICH: intracerebral hematoma; ICU: intensive care unit; IQR: interquartile range; M: Glasgow Coma Scale motor score; n: number of patients; PO1: post-operative day 1; SAH: aneurysmal subarachnoid haemorrhage; SD: standard deviation; TBI: traumatic brain injury; yrs: years.

**Fig. 2:**
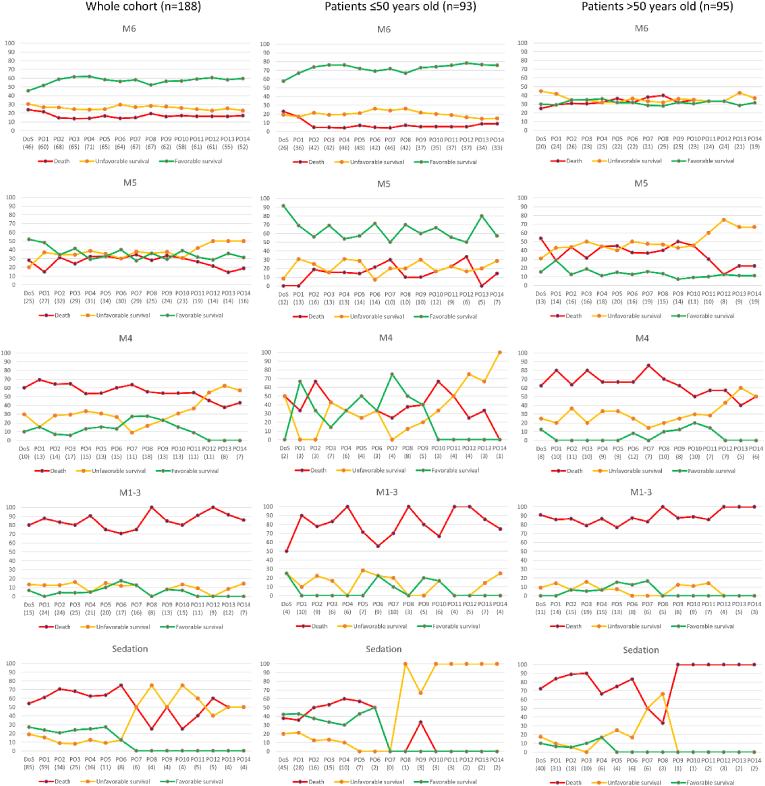
Relationship between one-year outcome and post-operative Glasgow Coma Scale motor scores during the first 14 days after decompressive craniectomy (DC) of 188 consecutive adult patients undergoing DC for various pathologies. DoS, day of surgery; M, Glasgow Coma Scale motor score; n, number of patients; PO, post-operative day; Sedation, sedated patients without recorded motor score. The numbers between parentheses below the x-axes refer to the number of patients on the respective post-operative day with the respective Glasgow Coma Scale motor score. Favourable survival was defined as Glasgow Outcome Scale score 4/5, unfavourable survival was defined as Glasgow Outcome Scale score 2/3.

Fifty-seven out of 108 M6 patients were functionally independent at one-year follow-up (53 %), versus only 4 % of M < 6 patients (p < 0.001). Fig. 2 presents the relation between the GCS M-scores during the first 14 post-DC days and long-term outcome. For each post-operative day, chances of functional independence in GCS M6 patients varied between 50 % and 60 %. From the day of surgery up to post-DC day 14, a GCS M6 score was a significant predictor of functional independence, with an odds Ratio (OR) ranging from 2.1 (95 % confidence interval (CI) 1.0–4.3) on the day of surgery to 13.5 (95 % CI 4.3–42.8) on post-DC day 12. The chance of favourable survival in the 45 patients who regained consciousness and then experienced neurological deterioration during the remainder of the first two post-DC weeks was 44 %, versus 60 % in patients without subsequent neurological deterioration and transferred awake patients (p = 0.09).

### 3.2 M6 patients ≤50 years old versus M6 patients >50 years old

Chances of functional independence in patients scoring M6 on any of the first 14 post-DC days were significantly higher in patients ≤50 years old than in patients >50 years old (71 % versus 27 %, p < 0.001). This difference was statistically different from post-DC day 1, with an OR ranging from 4.8 (95 % CI 1.6–14.5) on post-DC day 6–8.1 (95 % CI 2.4–27.8) on post-DC day 13. An inverse relation was observed for unfavourable survival (17 % versus 33 %, p < 0.05) and mortality (11 % versus 40 %, p < 0.001), with a median survival of 43 days (IQR 4–59) in GOS 1 M6 patients ≤50 years old, versus 53 days (IQR 24–116) in GOS 1 M6 patients >50 years old. Table 3 lists the causes of death among GOS 1 M6 patients, categorised by age groups. In both age groups, the majority of patients died following withdrawal of ventilation or following restriction of treatment (‘do not resuscitate’, DNR) because of neurological deterioration to poor GSC scores and presumed poor prognosis. While comparing M6 patients ≤50 years old versus M6 patients >50 years old (Table 4), M6 patients >50 years old tended to be more affected (albeit statistically non-significant), with more ischemic strokes (49 % versus 40 %) and fewer cerebral venous thrombosis (7 % versus 13 %) as underlying pathology, fewer patients with a pre-DC GCS M6 score (22 % versus 32 %), more additional surgeries following initial DC (33 % versus 24 %), and more (non-transferred) patients deteriorating neurologically (67 % versus 51 %). Withdrawal of ventilation because of presumed poor prognosis following neurological deterioration tended to occur more often among M6 patients >50 years old (13 % versus 5 %, p = 0.11), but at comparably low M scores as in M6 patients ≤50 years old (Table 3). Whether DNR was more often applied in older M6 patients compared to younger M6 patients could not be calculated, since we did not have access to the records of referring hospitals, nursing homes and rehabilitation clinics.

**Table 3:** Causes of death and length of survival of 25 patients undergoing decompressive craniectomy scoring GCS M6 during the first 14 post-operative days, categorised by age-groups ≤50 years old and >50 years old.

Cause of death	≤50 years old (n = 7)	>50 years old (n = 18)
Following withdrawal of ventilation b/o poor GCS		
- M1, n (days survival)	2 (2, 4)	4 (3, 4, 17, 26)
- M2, n (days survival)	1 (4)	2 (4, 30)

Following restriction of treatment (DNR) b/o poor GCS		
- M1, n (days survival)	1 (59)	3 (67, 79, 160)
- M3, n (days survival)		1 (27)
- M4, n (days survival)		1 (48)
- M5, n (days survival)	2 (43, 224)	1 (34)
- ‘comatose’ (days survival)		1 (212)

Pneumosepsis, n (days survival)	1 (56)	1 (120)

Glioblastoma, n (days survival)		1 (114)

Unknown, n (days survival)		3 (58, 72, 167)

b/o: because of; DNR: ‘do not resuscitate’; GCS: Glasgow Coma Scale; M: Glasgow Coma Scale motor score; n: number of patients.

**Table 4:** Characteristics of 108 patients undergoing decompressive craniectomy scoring GCS M6 during the first 14 post-operative days, categorised by age-groups ≤50 years old and >50 years old.

	≤50 years old	>50 years old	p-value
n (male/female)	63 (29/34)	45 (23/22)	
Indication for DC			
Ischemic stroke, n (%)	25 (40)	22 (49)	0.23
TBI, n (%)	7 (11)	4 (9)	
SAH, n (%)	10 (16)	7 (16)	
ICH, n (%)	9 (14)	6 (13)	
CVT, n (%)	8 (13)	3 (7)	0.25
Other, n (%)	4 (6)	3 (7)	
Neurologic condition pre-DC			
M6, n (%)	20 (32)	10 (22)	0.19
M5, n (%)	16 (25)	15 (33)	
M ≤ 4, n(%)	9 (14)	10 (22)	
Intubated & sedated, n (%)	17 (27)	10 (22)	
Additional surgery after DC, n (%)	15 (24)	15 (33)	0.19
Neurological deterioration following M6 in first 14 days post-DC, n (%)∗	25 (51)	20 (67)	0.13
ICU stay, days, median (IQR)	3 (1–7)	7 (3–15)	<0.001
Hospital stay, days, median (IQR)	27 (18–45)	33 (22–51)	
Withdrawal of ventilation, n (%)	3 (5)	6 (13)	0.11

CVT: cerebral venous thrombosis; DC: decompressive craniectomy; ICH: intracerebral hematoma; ICU: intensive care unit; IQR: interquartile range; M: Glasgow Coma Scale motor score; n: number of patients; PO1: post-operative day 1; SAH: aneurysmal subarachnoid haemorrhage; SD: standard deviation; TBI: traumatic brain injury; yrs: years; ∗14 awake patients ≤50 years old and 14 awake patients >50 years old were transferred to referring hospitals of rehabilitation clinics, without further recorded M scores. In one awake patient >50 years old at PO7, nu further M scores could be retrieved.

### 3.3 Regaining consciousness and outcome among the different causes for DC

**Fig. 3:**
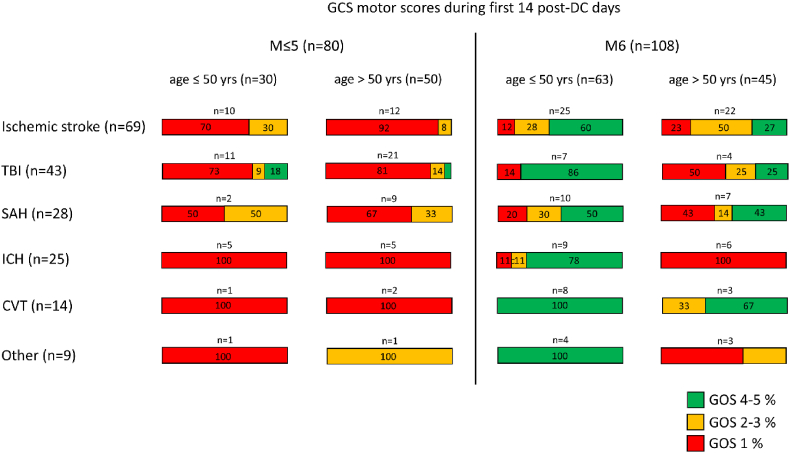
Relationship between one-year outcome and regaining consciousness during the first 14 days after decompressive craniectomy (DC) of 188 consecutive adult patients, when assorted for underlying cause for DC and age. CVT, cerebral venous thrombosis; GCS, Glasgow Coma Scale; GOS, Glasgow Outcome Scale; ICH, intracerebral hematoma; M, Glasgow Coma Scale motor score; n, number of patients; SAH, aneurysmal subarachnoid haemorrhage; TBI, traumatic brain injury; yrs, years.

The proportion of patients regaining consciousness during the first 14 days post-DC was highest in CVT (79 %), followed by brain infection/tumour (78 %), ischemic stroke (68 %), SAH (61 %), ICH (60 %) and TBI (26 %). The median day at which patients regained consciousness was the day of surgery for ischemic stroke (IQR 0–1) and brain infection/tumour (IQR 0–8), post-DC day 1 for CVT (IQR 0–2) and ICH (IQR 1–3), and post-DC day 2 for SAH (IQR 0.5–3) and TBI (IQR 2–9). The chance of long-term functional independence in M6 patients varied depending on the underlying pathology, ranging from 91 % for CVT patients to 45 % for ischemic stroke patients (Table 2). Fig. 3 summarises the correlation between regaining consciousness during the first 14 days post-DC and long-term outcome when assorted for underlying cause for DC and age. Three TBI patients (aged 19, 27 and 56 years old) did not regain consciousness during this time-frame but did survive independently at long-term follow-up (GOS 4).

### 3.4 Transferred versus non-transferred patients

A sub-analysis in the group of transferred patients showed that last-recorded GCS M-scores before transfer were M6 (71 %), M5 (24 %), M4 (2 %) and M2 (2 %). At one year follow-up, functional independence was significantly higher compared to non-transferred patients (56 % versus 25 %, p < 0.001). One-year functional independence in the 34 transferred M6 patients was 65 %; 17 out of 20 transferred M6 patients ≤50 years old survived favourably, versus 5 out 14 transferred M6 patients >50 years old (85 % versus 36 %, p < 0.01).

## 4 Discussion

We retrospectively analysed the one-year neurologic outcome of adult patients undergoing decompressive craniectomy for various pathologies and its relation with recovery of consciousness during the first 14 post-operative days. Almost 60 % of all patients regained consciousness during the first two weeks, and approximately half of them were functionally independent at one year follow-up. This is in sharp contrast with only 4 % functional independence in patients not regaining consciousness in the first two weeks following DC. Chances of functional independence in patients scoring M6 on any of the first 14 post-DC days were significantly higher in patients ≤50 years than in patients >50 years old (71 % versus 27 %, p < 0.001).

### 4.1 Literature on post-operative GCS scores and prognosis

Although post-operative GCS scores are routinely measured in all neurosurgical centers across the world, data in the literature on the relation between post-DC neurological scores and long-term outcome is extremely scarce. Thus far, only two articles reported data on the use of GCS scores as a potential prognostic factor (Chen et al., 2007; Ecker et al., 2011). Firstly, Chen et al. (2007) reported significantly more favourable outcome (defined as a Barthel index ≥60) at 12 months follow-up among 34 out of 60 malignant stroke patients who regained consciousness within 7 days after DC, compared to the 26 patients who had not regained consciousness by then (79 % versus 7 %, p < 0.0001). In our sub-cohort of 69 malignant stroke patients, we observed similar findings: 50 % of 36 patients who regained consciousness by post-DC day 7 survived favourably, compared to only 9 % of 33 patients who had not regained consciousness by then (p < 0.001). Secondly, Ecker et al. (2011) reported on outcome of American soldiers who underwent DC during the Iraqi and Afghan wars and who were subsequently transported to the United States of America (USA) for rehabilitation. In favourably surviving patients (GOS 4 or 5 at 1–5 years follow-up), mean GCS scores upon arrival in the USA were significantly higher (8 versus 4, p < 0.05) than in patients who eventually survived unfavourably (GOS 2 or 3) or died. However, it was not mentioned on what post-DC day patients arrived in the USA.

### 4.2 Post-DC counselling of family members and patients

How should we inform family members and patients about the prognosis during post-surgical counselling? The results of the current cohort may serve as a ‘guide’ during these uncertain times: very few patients who remained unconscious within the first 14 post-DC days survived independently, whereas functionally independent long-term survival in patients ≤50 years old with post-operative GCS M6 scores is very likely. However, much more caution is warranted in M6 patients >50 years old.

### 4.3 Limitations of the study

The methodology of the current cohort has several limitations. Firstly, data analysis was performed retrospectively, and a matched cohort of control patients who did not undergo DC was not available for analysis. Secondly, the data was relatively old (2006–2014). Setting up a more recent cohort is relevant, especially since the distribution of underlying causes for DC may have changed between 2014 en 2025; ischemic stroke was in the current cohort the leading cause for DC (37 %), but the introduction of endovascular thrombectomy has significantly reduced the number of annually performed craniectomies for ischemic stroke in our daily clinical practice since. Thirdly, we defined ADL independency at one year follow-up as favourable survival, but we did not measure long-term quality of life; ADL (in)dependency as primary outcome measure does not imply whether outcome is acceptable or not for an individual patient. We showed in previous work that good mental quality of life may occur in DC patients who remain ADL-dependent (van Middelaar et al., 2015). On the other hand, many ADL-independent survivors of acquired brain injury suffer from debilitating cognitive/emotional impairments and utilise different coping strategies, which greatly influence their quality of life (Cardile et al., 2024). Our current data may thus only serve to inform patients and family members about the chances of recovery of functional independence. Fourthly, we used a GCS M6 score as a depicter of recovery of consciousness, whereas nowadays the Coma Recovery Scale-Revised (CRS-R) is considered as a much more reliable test to measure consciousness (Giacino et al., 2004). However, CRS-R measurements are more elaborative and time-consuming, and therefore less applicable in daily clinical practice. Fifthly, this large cohort contained patients with different underlying pathologies, and outcome in M6 patients varied considerably among pathologies. In Fig. 3, we illustrated the correlation between regaining consciousness and outcome when assorted for underlying cause for DC and age, but one should be aware that the large cohort is then split up in many small(er) subgroups, hampering proper statistical analysis. Sixthly, 41 out of 188 patients did not have a full data set of M-scores throughout the 14 days post-DC time frame because of (awake) transfer to referring hospitals or rehabilitation clinics. Seventhly, we used clinician's notes at one-year post-DC to retrospectively assess outcome according to the GOS, whereas the standard way to assess GOS scores is through a validated structured interview (Wilson et al., 1998). Finally, another important limitation may have been the so-called self-fulfilling prophecy, i.e. the tendency to restrict treatment selectively in patients with certain characteristics presumed to predict unfavourable outcome (Kirkman et al., 2013; Zahuranec et al., 2007). Indeed, the majority of M6 patients in the current cohort died of respiratory failure following withdrawal/restriction of treatment applied because of subsequent neurological deterioration and presumed low-GCS-score-related prognosis. Would some of these patients still be alive at long-term follow-up if active treatment had been continued? And, if yes, in what condition? These questions should be kept in mind when interpreting the current data. Furthermore, more insight in the overall occurrence of treatment restrictions, insight in the timepoints at which treatment is restricted, and insight in the specific patient characteristics upon which clinicians decide to restrict treatment, may be gathered by the creation of a nationwide prospective DC outcome registry. In our level-1 university medical centre, DC was performed (on average) only twice per month during this 8 year time-frame. A nationwide registry would allow for analysis of considerably larger patient cohorts. To cover the whole long-term chain of care, it is crucial to include rehabilitation clinic/nursing home physicians as well as (neuro)psychologists in such registry.

## 5 Conclusions

Long-term functional outcome of DC patients differed considerably when assorted for early recovery of consciousness. On each post-operative day, recovery of consciousness (GCS M6 score) was a significant predictor of functional independence, and very few patients who remained unconscious within the first 14 post-DC days survived independently. However, chances of functional independence in M6 patients were significantly lower in patients >50 years old compared to patients ≤50 years old. Our results may serve to better inform family members and patients during post-DC counselling.

## Author contribution

Study concept (PvdM), data collection (JG, PvdM), first draft manuscript (JG, TG, DV, PvdM), editing and final review (JG, TG, DV, BC, BK, JC, JH, WV, PvdM)

## Disclosure statement

No potential conflict of interest was reported by the authors.

## Funding

No specific funding was allocated for this study.

## Declaration of competing interest

The authors declare that they have no known competing financial interests or personal relationships that could have appeared to influence the work reported in this paper.
